# Admission albumin and C-reactive protein-to-lymphocyte ratio are associated with MRI-defined abscess in children with acute hematogenous osteomyelitis: a two-center retrospective study

**DOI:** 10.3389/fcimb.2026.1822701

**Published:** 2026-07-10

**Authors:** Chaochen Zhao, Zhiye Guan, Chenghui Ke, Xiaodong Wang, Yinxian Chen, Lihua Zhao

**Affiliations:** 1 Department of Orthopedics, Shanghai Children’s Hospital, School of Medicine, Shanghai Jiao Tong University, Shanghai, China; 2 Department of Orthopedics, Children’s Hospital of Soochow University, Suzhou, Jiangsu, China

**Keywords:** acute hematogenous osteomyelitis, albumin, CRP-to-lymphocyte ratio, MRI-defined abscess, pediatric infection

## Abstract

**Introduction:**

Intramedullary and subperiosteal abscesses are clinically relevant MRI-defined phenotypes in children with acute hematogenous osteomyelitis (AHO). This study examined admission-phase markers associated with MRI-defined abscess and constructed an exploratory pre-MRI risk-stratification model.

**Methods:**

This two-center retrospective cohort study included 105 children with AHO who underwent MRI within 72 hours of admission. Clinical variables and admission laboratory indices were analyzed using univariable analyses and multivariable Firth penalized logistic regression. Model performance was assessed using receiver operating characteristic analysis, calibration assessment, decision curve analysis, stratified cross-validation, and bootstrap optimism correction.

**Results:**

MRI-defined abscess was present in 45 children (42.9%). Lower albumin and higher CRP-to-lymphocyte ratio were independently associated with MRI-defined abscess, whereas fever at admission was retained as a prespecified clinically relevant covariate rather than interpreted as an independent predictor. The final model showed modest discrimination (apparent AUC, 0.711; bootstrap 95% CI, 0.624-0.826; optimism-corrected AUC, 0.689). At the exploratory Youden-derived cutoff of 0.456, sensitivity was 0.600 and specificity was 0.767.

**Conclusion:**

Admission albumin and CRP-to-lymphocyte ratio are readily available markers associated with MRI-defined abscess and may support early identification of children who warrant heightened attention to timely MRI and multidisciplinary review. The model requires external validation and should complement, not replace or delay, clinical assessment, MRI, and surgical evaluation.

## 1 Introduction

Acute hematogenous osteomyelitis (AHO) is a common pediatric musculoskeletal infection and can be associated with substantial morbidity (Woods et al., 2021). It often has an acute presentation and may progress quickly; delays in diagnosis or suboptimal early management can lead to bone destruction, a prolonged clinical course, and persistent functional sequelae (Alhinai et al., 2020). In children, the vascular architecture of the growing skeleton and the features of the periosteum favor initial infection in the metaphysis of long bones, with subsequent spread into the medullary cavity and/or along the subperiosteal space (McNeil, 2020). These routes of extension contribute to the variability in imaging findings and clinical presentation. As pathogen patterns and treatment approaches have evolved, emphasis has increasingly shifted toward early risk assessment and phenotype-guided management to support timely decisions in the initial stage of illness (Alhinai et al., 2020; Stephan et al., 2024).

Among complicated presentations of AHO, intramedullary and subperiosteal abscesses are clinically relevant MRI-defined phenotypes (Woods et al., 2021; Hartman et al., 2022). Their presence often indicates a drainable collection and may affect antimicrobial duration, surgical drainage decisions, inpatient monitoring, and follow-up intensity (Connolly et al., 2007; Zhao et al., 2024).

Magnetic resonance imaging (MRI) can detect bone marrow edema early, define the extent of disease, and identify intramedullary and subperiosteal abscesses; for these reasons, it is commonly used for phenotypic assessment in pediatric AHO (Chan et al., 2024; Shet et al., 2022; Habre et al., 2022). However, many guidelines and reviews also note that MRI is not always available in a timely manner. In primary or resource-limited settings, access may be constrained by scanner availability, scheduling delays, transfer requirements, and limited capacity for pediatric sedation or anesthesia. Even in tertiary centers, the need for sedation may delay imaging (Marin et al., 2024). When MRI is unavailable or cannot be obtained promptly, alternative imaging strategies (such as ultrasound, computed tomography, or radionuclide studies) are often used to assess for collections and estimate disease extent (Hosokawa et al., 2024). In practice, initial physical examination and routine laboratory tests do not consistently distinguish uncomplicated AHO from an MRI-defined abscess phenotype, creating uncertainty about which children should be prioritized for urgent MRI, referred early, or monitored more closely with early surgical input.

The presence of an abscess may reflect pathogen-related factors, local anatomy, and the host inflammatory response. Systemic biomarkers should therefore be interpreted as clinical correlates of this phenotype, not as direct mechanistic evidence of abscess development. Admission laboratory markers may still be useful before MRI results are available, particularly when imaging or sedation capacity is constrained (Wiedermann, 2021; Shi et al., 2023; Hou et al., 2025). Evidence linking these early markers to MRI-defined abscess phenotypes in pediatric AHO remains limited (Alhinai et al., 2020; Zhao et al., 2024).

We therefore aimed to identify admission-phase clinical and laboratory markers associated with MRI-defined intramedullary and/or subperiosteal abscess in pediatric AHO and to derive a parsimonious pre-MRI risk-stratification model that could inform prioritization of imaging or multidisciplinary review.

## 2 Materials and methods

This study was approved by the institutional review boards of the two participating hospitals (approval numbers: 2021KS024 and 2025R044-E01). Given the retrospective design and use of de-identified data, the requirement for informed consent was waived. The study was conducted in accordance with the Declaration of Helsinki.

### 2.1 Study design, setting, and study subjects

We conducted a two-center retrospective cohort study. Consecutive children admitted to the orthopedic departments of Shanghai Children’s Hospital and the Children’s Hospital of Soochow University between January 2015 and December 2024 with AHO were screened. Both participating institutions are tertiary pediatric referral centers equipped with two MRI scanners, which facilitated timely MRI scheduling and relatively uniform ascertainment of the MRI-defined outcome.

Patients were eligible if they met all of the following criteria: (1) diagnosis of AHO; (2) MRI performed within 72 hours of admission; (3) symptom onset within 14 days prior to hospitalization and no antibiotic exposure before the first blood sampling; and (4) age between 1 month and 18 years. Exclusion criteria were: (1) underlying chronic or systemic diseases (e.g., hematologic disorders, rheumatologic disease, cardiovascular disease, malignancy, or other chronic conditions); (2) osteomyelitis related to open fractures or postoperative infection; and (3) incomplete or unavailable medical records.

### 2.2 Clinical data collection and laboratory measurements

Demographic and baseline clinical variables were extracted from medical records, including sex, age, infection site, and body temperature at admission. Laboratory tests obtained within 24 hours of admission included WBC, CRP, ESR, neutrophil count, lymphocyte count, and serum albumin. Fever at admission was defined as a temperature >38.5 °C. Two composite indices were calculated: the CRP-to-albumin ratio (CAR = CRP/albumin) and the CRP-to-lymphocyte ratio (CLR = CRP/lymphocyte count). These indices were included to combine CRP with albumin (a negative acute-phase reactant and proxy for nutritional status) and lymphocyte count (a marker of host immune status), thereby capturing inflammatory burden and host response using routinely available tests. All baseline blood samples were collected prior to initiation of antibiotic therapy.

### 2.3 Imaging review and definition of MRI-defined abscess

All MRI examinations were acquired on 3.0T scanners and interpreted during routine clinical care by institutional radiologists. For this study, two pediatric orthopedic surgeons independently re-reviewed the images while blinded to laboratory-derived model predictors. Discrepancies were resolved by consensus; unresolved cases were adjudicated by a pediatric radiologist as the third reviewer. A formal interobserver reliability coefficient was not available in this retrospective dataset.

Primary outcome: MRI-defined intramedullary and/or subperiosteal abscess, defined as a focal and relatively well-demarcated fluid cavity with a T2-weighted/STIR hyperintense core and corresponding T1-weighted hypointensity, consistent with a discrete collection (Alaia et al., 2021). Diffuse marrow or soft-tissue edema without a definable focal fluid cavity was not classified as abscess. Abscess size, volume, number, and anatomic extent were not systematically quantified in this retrospective study.

### 2.4 Anatomic classification of abscesses

For patients with MRI-defined abscess, collections were categorized by anatomic compartment when the imaging report or chart review provided sufficient detail. Intramedullary abscess was defined as a fluid collection within the medullary cavity; subperiosteal abscess was defined as a fluid collection between the cortical surface and elevated periosteum. Isolated soft-tissue abscesses without intramedullary or subperiosteal involvement, and inflammatory infiltration/cellulitis without a discrete fluid cavity, were not counted as the primary outcome, consistent with standardized MRI nomenclature (Alaia et al., 2021).

### 2.5 Diagnostic criteria and treatment strategy

The diagnosis of AHO was established primarily by microbiological confirmation, defined as identification of a pathogen from blood culture or culture of aspirated pus/tissue. For culture-negative cases, AHO was considered when compatible clinical features were present (e.g., fever, localized swelling, pain, or restricted motion), accompanied by elevated inflammatory markers and imaging findings supportive of infection (radiography and/or MRI), with clear clinical improvement within 48 hours after initiation of antibiotic therapy.

All children with suspected AHO underwent blood cultures on admission and received empirical intravenous first- or second-generation cephalosporins. Antimicrobial therapy was subsequently adjusted according to culture results and susceptibility testing when available. Decisions regarding surgical intervention were made by the treating team based on the clinical course, imaging findings, and response to antimicrobial therapy.

### 2.6 Statistical analysis

All statistical analyses were performed using IBM SPSS Statistics version 26.0 (IBM Corporation, Armonk, NY, USA) and R software version 4.5.1 (R Foundation for Statistical Computing, Vienna, Austria). Normally distributed continuous variables were expressed as mean ± standard deviation, and non-normally distributed variables were reported as median (P25, P75). Categorical variables were presented as frequencies and percentages. Between-group comparisons used the independent-samples t-test, Mann-Whitney U test, chi-square test, or Fisher’s exact test, as appropriate. Univariable logistic regression described crude associations between prespecified candidate predictors and MRI-defined abscess, but it was not used as the sole basis for final predictor selection.

Because the sample size was limited, Firth penalized logistic regression was used to reduce small-sample bias and obtain more stable coefficient estimates. Fever at admission was retained as a prespecified clinically relevant bedside covariate because it showed a borderline univariable association and is directly relevant to early clinical assessment; it was not interpreted as an independent predictor after adjustment. Results are reported as odds ratios with 95% confidence intervals. Discrimination was assessed using AUC, cross-validation, and bootstrap optimism correction. Calibration was assessed visually, by the Hosmer-Lemeshow test, and by the Brier score; these results were interpreted with caution because calibration estimates have limited precision in small samples. The Youden cutoff was reported as an exploratory threshold derived and evaluated in the same dataset. Decision curve analysis was used to examine net benefit across clinically plausible threshold probabilities for prioritizing MRI or multidisciplinary review, not for excluding abscess. The study was reported in accordance with TRIPOD principles where applicable to a retrospective internally validated prediction model.

To make predictor selection more transparent, five clinically plausible models were compared: the primary model (fever + albumin + CLR), model A (fever + CRP + albumin), model B (fever + CAR), model C (fever + CLR), and model D (fever + CRP + lymphocyte count + albumin). Models were compared using penalized AIC, apparent AUC, optimism-corrected AUC, Brier score, and cross-validated AUC. Calibration was also assessed using the calibration intercept and calibration slope. The intercept quantifies systematic over- or underestimation (ideal value, 0), and the slope quantifies the spread of predicted probabilities (ideal value, 1; slope < 1 suggests overfitting). Given the modest sample size, these indices were interpreted descriptively alongside visual calibration and the Brier score.

## 3 Results

### 3.1 General information

The cohort included 105 children with acute hematogenous osteomyelitis who underwent MRI (66 males and 39 females). Forty-five children (42.9%) had MRI-defined intramedullary and/or subperiosteal abscess, and 60 (57.1%) did not. Because retrospective MRI reports did not consistently use standardized compartment-level terminology to distinguish isolated intramedullary abscess, isolated subperiosteal abscess, combined lesions, and soft-tissue extension, quantitative subtype frequencies were not considered reliable for reporting. The median age was 8.25 years (IQR, 3.58-10.58). The femur was the most frequently involved bone, followed by the tibia and pelvis.

### 3.2 Systematic comparison between abscess and non-abscess groups

Comparisons between the abscess and non-abscess groups showed no significant differences in sex, age, time from symptom onset to presentation, or the presence of suppurative arthritis (all P > 0.05). Similarly, white blood cell count (WBC), erythrocyte sedimentation rate (ESR), and neutrophil count did not differ between groups (all P > 0.05). The proportion of fever at admission was higher in the abscess group than in the non-abscess group (62.2% vs. 43.3%), although the difference did not reach statistical significance (P = 0.055).

By contrast, inflammatory and derived indices differed between groups. The abscess group had higher C-reactive protein (CRP) levels than the non-abscess group (median 63.5 vs. 41.6 mg/L, P = 0.003) and lower serum albumin (median 40.3 vs. 42.6 g/L, P = 0.017). Consistently, both CAR and CLR were higher in the abscess group (both P = 0.002) (**Table 1**).

**Table 1:** Baseline characteristics and admission laboratory findings in children with acute hematogenous osteomyelitis, stratified by MRI-defined abscess.

Variables	Abscess group (n = 45)	Non-abscess group (n = 60)	P
Sex, n (%)			0.268
Male	31 (68.9)	35 (58.3)	
Female	14 (31.1)	25 (41.7)	
Age (years), Median (IQR)	8.17 (4.58, 9.92)	8.50 (2.79, 11.20)	0.928
Fever at admission, n (%)	28 (62.2)	26 (43.3)	0.055
Suppurative arthritis, n (%)	9 (20.0)	10 (16.7)	0.661
Symptom duration before admission (days), Median (IQR)	6.00 (3.00, 7.00)	6.00 (4.00, 7.00)	0.543
WBC (×109/L), Median (IQR)	12.90 (9.28, 15.40)	11.00 (8.50, 14.60)	0.398
CRP (mg/L), Median (IQR)	63.50 (40.50, 116.00)	41.60 (13.10, 77.00)	0.003
ESR (mm/h), Median (IQR)	47.00 (30.00, 77.00)	44.50 (24.00, 66.00)	0.571
Neutrophils (×10^9^/L), Median (IQR)	8.27 (6.76, 11.10)	7.36 (4.49, 10.00)	0.112
Lymphocytes (×10^9^/L), Median (IQR)	1.98 (1.17, 3.40)	2.66 (1.90, 3.58)	0.057
Albumin (g/L), Median (IQR)	40.30 (36.60, 43.00)	42.60 (39.30, 44.40)	0.017
CAR, Median (IQR)	1.61 (1.02, 3.13)	0.96 (0.32, 2.05)	0.002
CLR, Median (IQR)	30.30 (13.50, 78.30)	14.50 (4.03, 36.00)	0.002

Values are expressed as median (interquartile range) or n (%). Continuous variables were compared using the Mann-Whitney U test, and categorical variables were compared using the chi-square test or Fisher’s exact test, as appropriate. Sex refers to biological sex recorded in the medical record. WBC, white blood cell count; CRP, C-reactive protein; ESR, erythrocyte sedimentation rate; ALB, albumin; CAR, CRP-to-albumin ratio; CLR, CRP-to-lymphocyte ratio.

Microbiological data were reviewed descriptively because pathogen variables may be influenced by sampling practice as well as disease severity. Blood culture was obtained in 90 children (85.7%) and was positive in 19 children (18.1% of the full cohort; 21.1% of those cultured), all with *Staphylococcus aureus*. *S. aureus* infection was defined as *S. aureus* identified from blood culture or pus/tissue culture. *S. aureus* infection was more frequent in the abscess group than in the non-abscess group (27/45, 60.0% vs. 18/60, 30.0%; Fisher’s exact P = 0.003). MRSA was identified in 14 children (13.3%) and did not differ significantly between groups (8/45, 17.8% vs. 6/60, 10.0%; Fisher’s exact P = 0.263). Because microbiological confirmation depends on blood culture, pus/tissue culture, prior antibiotics, and surgical sampling, pathogen variables were not included in the primary prediction model.

Clinical management outcomes were consistent with greater disease severity in the MRI-defined abscess group. Surgical intervention was more frequent among children with abscess than among those without abscess (34/45, 75.6% vs. 17/60, 28.3%; P < 0.001). The abscess group also had a longer median hospital stay (24 vs. 17 days), higher mean peak CRP (104.1 vs. 61.7 mg/L), and more frequent persistent high fever (22/45, 48.9% vs. 13/60, 21.7%).

### 3.3 Univariable logistic regression analysis

Univariable logistic regression identified several candidate predictors of MRI-defined abscess (**Table 2**). Fever at admission showed a borderline association (OR, 2.154; 95% CI, 0.978–4.745; P = 0.057). CRP was positively associated with MRI-defined abscess (OR, 1.010 per mg/L; 95% CI, 1.002–1.017; P = 0.010), whereas albumin was inversely associated (OR, 0.884 per g/L; 95% CI, 0.801–0.976; P = 0.015). Both CAR (OR, 1.454; 95% CI, 1.096–1.930; P = 0.009) and CLR (OR, 1.012; 95% CI, 1.003–1.021; P = 0.010) were also positively associated with MRI-defined abscess. Lymphocyte count was not significantly associated with MRI-defined abscess (P = 0.112) (**Table 2**).

**Table 2:** Univariable logistic regression analyses of factors associated with MRI-defined abscess.

Variable	β	Standard Error	χ²	P	OR	95% CI
Fever at admission	0.767	0.403	3.625	0.057	2.154	0.978–4.745
CRP	0.010	0.004	6.552	0.010	1.010	1.002–1.017
Lymphocyte count	-0.202	0.127	2.532	0.112	0.817	0.637–1.048
ALB	-0.123	0.051	5.925	0.015	0.884	0.801–0.976
CAR	0.374	0.144	6.733	0.009	1.454	1.096–1.930
CLR	0.012	0.005	6.596	0.010	1.012	1.003–1.021

Univariable logistic regression was used to describe crude associations between prespecified candidate predictors and MRI-defined abscess. P < 0.05 was considered statistically significant. CRP, C-reactive protein; ALB, albumin; CAR, CRP-to-albumin ratio; CLR, CRP-to-lymphocyte ratio.

### 3.4 Nonlinearity and multicollinearity assessment

Before multivariable modeling, multicollinearity was assessed using variance inflation factors (VIFs) and tolerance, given the inherent dependency between CRP and its derived ratios. Marked collinearity was observed between CRP and CAR (VIF, 108.99 and 117.01, respectively; tolerance = 0.009), consistent with mathematical coupling and suggesting a high likelihood of unstable coefficient estimates if entered simultaneously. In contrast, collinearity was acceptable for CLR (VIF, 3.26), albumin (VIF, 2.81), and fever at admission (VIF, 1.12). Accordingly, CRP and CAR were not included in the same multivariable model. To reduce model instability, subsequent analyses prioritized clinically interpretable variables with acceptable collinearity, particularly CLR and albumin.

Restricted cubic spline analyses were then used to assess potential non-linear associations of albumin and CLR with abscess risk. No evidence of non-linearity was observed for either predictor (albumin: χ² = 0.68, P = 0.410; CLR: χ² = 0.48, P = 0.489), indicating approximately linear relationships within the observed range. Therefore, albumin and CLR were retained as linear terms in the multivariable model.

### 3.5 Multivariable logistic regression analysis

In the multivariable Firth penalized logistic regression, the overall model was statistically significant (likelihood ratio chi-square = 16.606, P < 0.001). Albumin remained independently associated with lower odds of MRI-defined abscess (OR, 0.886 per g/L; 95% CI, 0.796-0.982; P = 0.020), and CLR remained associated with higher odds (OR, 1.007 per unit; 95% CI, 1.001-1.017; P = 0.011). Fever at admission was retained as a clinically relevant covariate but was not independently associated after adjustment (OR, 1.879; 95% CI, 0.814-4.432; P = 0.140).

The final model was specified as: logit[P(MRI-defined abscess)] = 3.981 + 0.631 × fever at admission - 0.120 × albumin + 0.007 × CLR, where fever at admission was coded as 1 for temperature >38.5 °C and 0 otherwise (**Table 3**).

**Table 3:** Multivariable Firth penalized logistic regression for MRI-defined abscess.

Variable	β	Standard error	χ²	P	OR	95% CI
Fever at admission	0.631	0.428	2.173	0.140	1.879	0.814–4.432
ALB	-0.120	0.052	5.388	0.020	0.886	0.796–0.982
CLR	0.007	0.003	4.523	0.011	1.007	1.001–1.017
Intercept	3.981	2.130	3.492	0.066	—	—

Firth penalized logistic regression was applied to mitigate small-sample bias. The intercept is retained for model specification but is not clinically interpreted as an odds ratio. Fever was retained as a prespecified clinically relevant covariate; it was not independently associated after adjustment. ALB, albumin; CLR, C-reactive protein to lymphocyte ratio.

In exploratory comparisons of five prespecified candidate models, the primary model had the lowest penalized AIC (111.740), an apparent AUC of 0.711, an optimism-corrected AUC of 0.689, a Brier score of 0.206, and a cross-validated AUC of 0.664. Alternative models had higher penalized AIC values (113.550-130.810), lower optimism-corrected AUC values (0.664-0.669), higher Brier scores (0.214-0.223), and cross-validated AUC values of 0.619-0.638. The fever + albumin + CLR model was retained as the primary specification because it was parsimonious, clinically interpretable, and not affected by the severe collinearity seen when CRP and CAR were modeled together.

### 3.6 Model performance and internal validation

**Figure 1:**
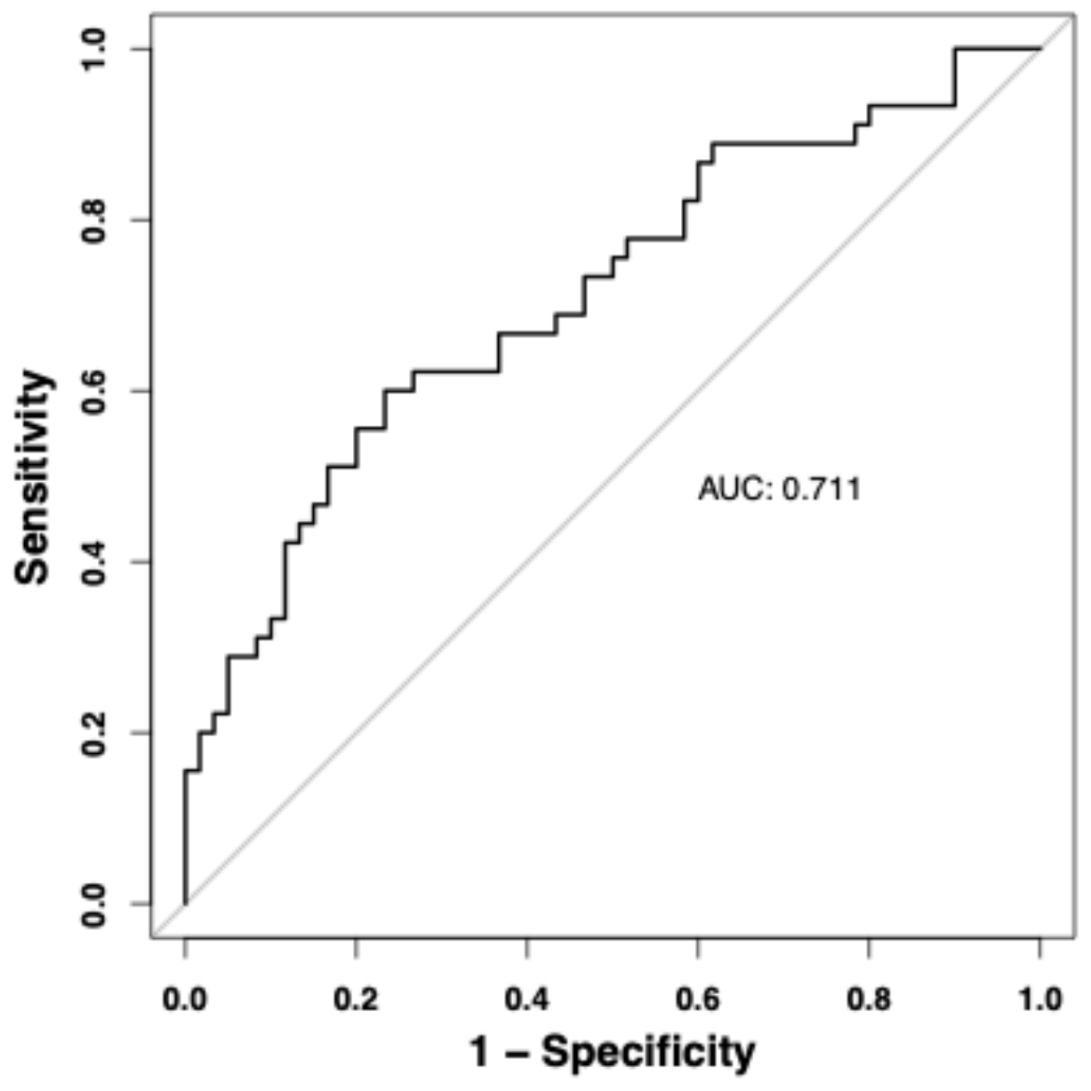
Receiver operating characteristic curve of the model for assessing MRI-defined abscess. The apparent AUC was 0.711, indicating modest discrimination; it should not be interpreted as diagnostic certainty.

Model performance was evaluated using 10-fold stratified cross-validation and bootstrap optimism correction. The cross-validated AUC was 0.664, and mean cross-validation accuracy was 0.677 +/- 0.183. The Firth penalized logistic regression model showed modest discrimination, with an apparent AUC of 0.711 (bootstrap 95% CI, 0.624-0.826) (**Figure 1**). The optimism-corrected AUC was 0.689, indicating modest internal performance after correction for overoptimism.

**Figure 2:**
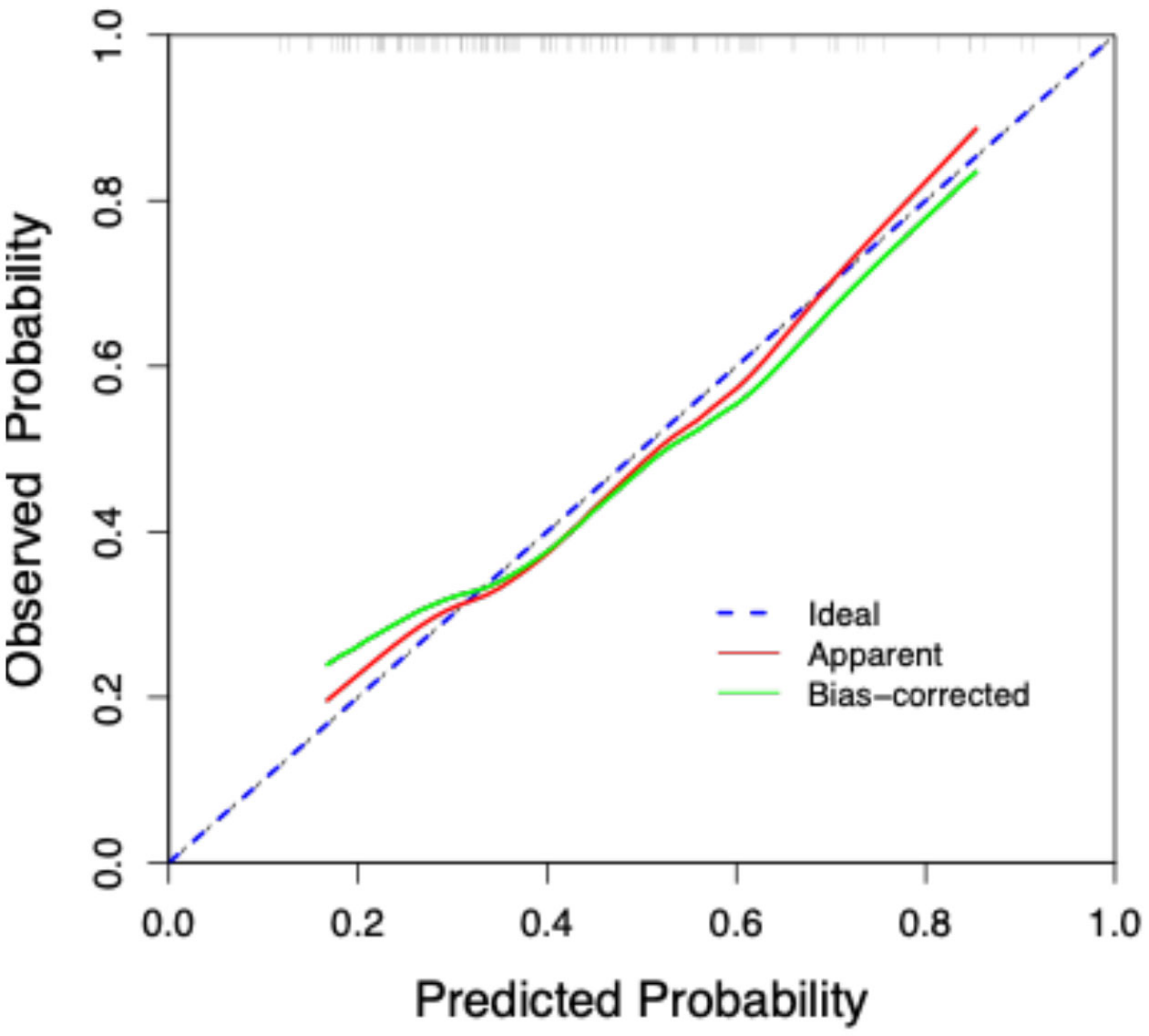
Calibration plot of the multivariable model. The x-axis indicates predicted probability and the y-axis indicates observed probability of MRI-defined abscess. Bias correction was performed using 1,000 bootstrap resamples. Calibration was assessed visually and should be interpreted cautiously because of the modest sample size.

**Figure 3:**
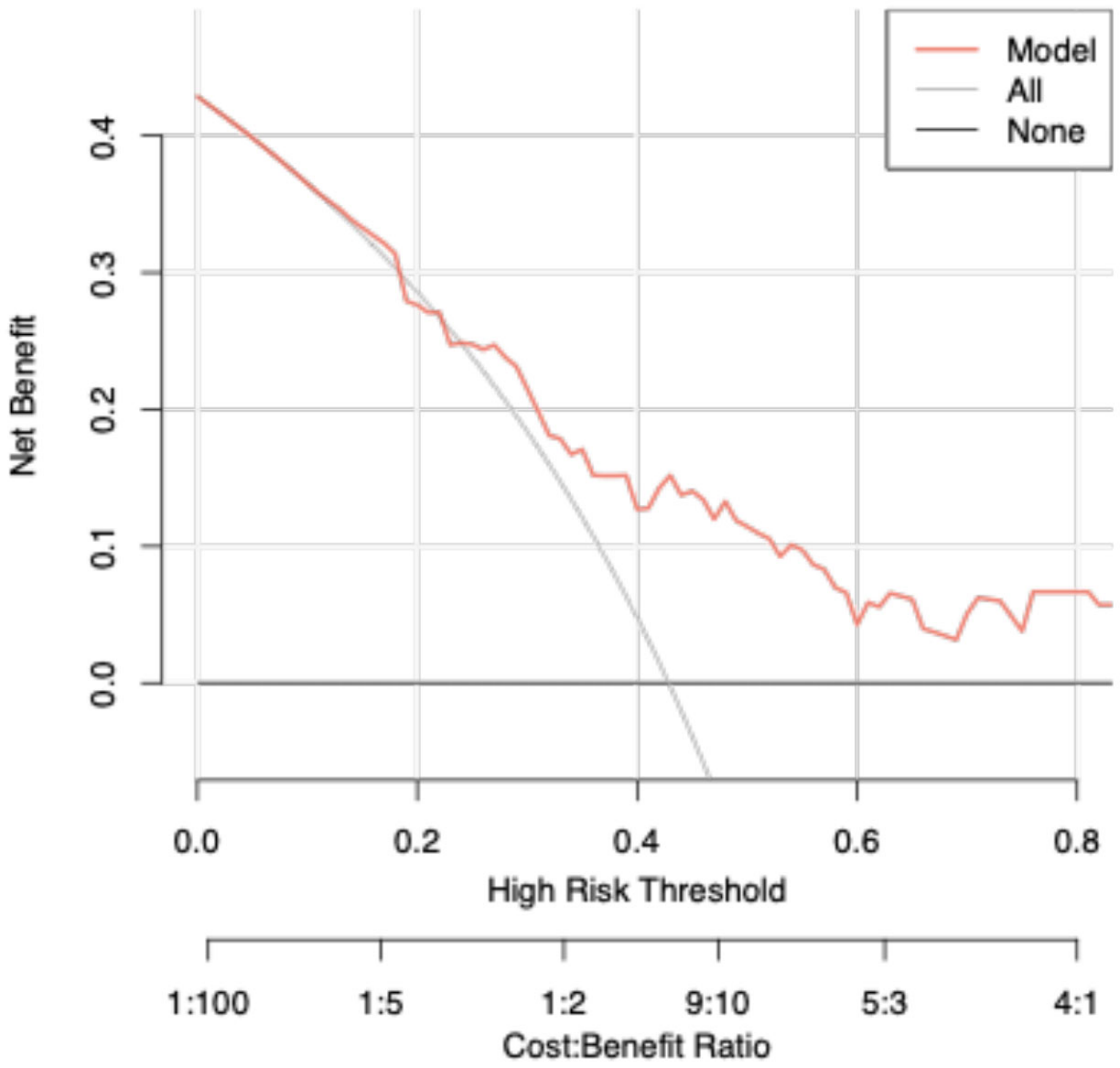
Decision curve analysis for the model. Net benefit is plotted against threshold probability. Potential net benefit is relevant only within clinically plausible thresholds for prioritizing MRI or multidisciplinary review and does not justify deferring imaging or surgical evaluation.

The Youden-derived cutoff was 0.456. At this exploratory threshold, sensitivity was 0.600 (95% CI, 0.444-0.742), specificity was 0.767 (95% CI, 0.641-0.862), PPV was 0.659 (95% CI, 0.494-0.797), and NPV was 0.719 (95% CI, 0.594-0.822) (**Table 4**). Calibration assessment showed no evidence of major miscalibration (Hosmer-Lemeshow P = 0.966). The apparent calibration intercept was -0.000 and the apparent calibration slope was 0.999, consistent with near-ideal apparent calibration in the development dataset. After 1,000 bootstrap resamples, the optimism-corrected calibration intercept was -0.018 and the optimism-corrected calibration slope was 0.892, suggesting mild overfitting (slope < 1), consistent with the optimism observed for the AUC. Because of the modest sample size, the Hosmer-Lemeshow test has limited power to detect miscalibration, and calibration findings should be interpreted cautiously. The Brier score was 0.206 (**Figure 2**). Decision curve analysis suggested possible net benefit across clinically plausible threshold probabilities for prioritizing MRI or early multidisciplinary review (approximately 0.25-0.60), but the model should not be used to rule out abscess or delay imaging or surgical evaluation (**Figure 3**).

**Table 4:** Receiver operating characteristic analysis of the final model for assessing MRI-defined abscess.

Variables	AUC (95% CI)	Cutoff value	Sensitivity (95% CI)	Specificity (95% CI)	PPV (95% CI)	NPV (95% CI)	Youden index	Accuracy
Model	0.711 (0.624-0.826)	0.456	0.600 (0.444-0.742)	0.767 (0.641-0.862)	0.659 (0.494-0.797)	0.719 (0.594-0.822)	0.367	0.695

AUC confidence intervals were derived using bootstrap resampling. The cutoff was selected by the Youden index (sensitivity + specificity - 1) and should be regarded as exploratory because it was derived and evaluated in the same dataset. AUC, area under the curve; PPV, positive predictive value; NPV, negative predictive value.

## 4 Discussion

In this two-center retrospective cohort of 105 children with pediatric AHO, lower admission ALB and higher CLR, together with fever at admission, identified children who were more likely to have an MRI-defined intramedullary and/or subperiosteal abscess. These findings suggest that routinely available admission biomarkers may provide clinically relevant risk-stratification information before or alongside MRI. However, the model should be interpreted cautiously because its internally validated performance was modest and because the study was conducted in two tertiary pediatric centers where MRI was obtained within 72 hours of admission.

The clinical relevance of this MRI-defined phenotype is supported by the observed differences between children with and without abscess. Children with MRI-defined abscess had more frequent *S. aureus* infection, higher rates of surgical intervention, longer hospitalization, higher peak CRP, more persistent high fever, and more frequent complex disease course. These findings are consistent with the concept that MRI-defined abscess represents a more severe and management-relevant phenotype in pediatric AHO (Hunter and Baker, 2023; Johnston et al., 2017). Nevertheless, these microbiological and management variables were considered descriptive rather than primary model predictors because they may be influenced by imaging access, surgical sampling, culture yield, and clinician decision-making.

The retained predictors are clinically plausible, but they should not be overinterpreted mechanistically. Albumin is a negative acute-phase reactant and may also reflect nutritional-inflammatory reserve, vascular permeability, metabolic stress, and overall systemic illness (Mantovani and Garlanda, 2023; Evans et al., 2021; Soeters et al., 2019; Wiedermann, 2021). Therefore, lower admission ALB in children with MRI-defined abscess may be interpreted as a pragmatic marker of greater systemic inflammatory burden or reduced physiological reserve rather than as a pathogen-specific or abscess-specific mechanism. This interpretation is consistent with broader infection and critical illness literature, but direct pediatric AHO-specific evidence remains limited.

CLR may provide complementary information because it integrates CRP-related inflammatory burden with lymphocyte count (Gabay and Kushner, 1999). A higher CLR may reflect a stronger acute-phase response together with relative lymphocyte suppression or redistribution during systemic inflammatory stress (Hotchkiss et al., 2013; Rubio et al., 2019; Zhong and Zhao, 2025; Drewry et al., 2014; Podd et al., 2023). In the present cohort, CLR was associated with MRI-defined abscess, whereas WBC count, neutrophil count, and ESR did not differ significantly between groups, suggesting that composite inflammatory indices may capture admission-phase information not reflected by conventional single markers (Shi et al., 2023; Hou et al., 2025). However, this retrospective study does not provide direct evidence of host-pathogen interaction, immune dysregulation, lymphocyte dysfunction, or local abscess biology. The biological interpretation of CLR should therefore be regarded as clinical context and hypothesis generation rather than evidence of a causal pathway.

Fever at admission also requires cautious interpretation. Although fever showed a borderline univariable association with MRI-defined abscess, it was not independently associated after adjustment. It was retained in the model because it is immediately available at presentation, clinically relevant to bedside assessment, and consistent with early severity evaluation in pediatric AHO, not because it should be considered an independent biomarker (McNeil et al., 2019).

From a model-performance perspective, the apparent AUC of 0.711 and optimism-corrected AUC of 0.689 indicate modest discrimination. At the exploratory Youden-derived cutoff of 0.456, specificity was higher than sensitivity, and the NPV was not sufficient to safely rule out abscess. Accordingly, the model should not be used to defer MRI, drainage, or surgical evaluation. Its potential role is limited to adjunctive risk stratification for prioritizing earlier MRI scheduling, sedation resources, or orthopedic/infectious disease review when several clinically stable children compete for limited resources (Vanderby et al., 2010).

The decision curve analysis should be interpreted within this restricted clinical use-case. The threshold probability range of approximately 0.25-0.60 does not represent a range for withholding MRI. Rather, it represents a clinically uncertain zone in which clinicians may reasonably prioritize earlier MRI or multidisciplinary review for otherwise stable children, while accepting that lower thresholds will increase false-positive prioritization to reduce the chance of missing a drainable collection. Children with concerning examination findings, persistent fever, rising inflammatory markers, sepsis, or poor early response to therapy require escalation regardless of model-predicted probability.

Several limitations merit consideration. First, the sample size and event count were modest (n = 105; 45 events), with an events-per-variable ratio of approximately 15 for three predictors. This ratio supports a compact model but does not support more complex specifications or stable subgroup analyses. The relatively wide confidence intervals reflect this uncertainty, and external validation remains necessary.

Second, inclusion required MRI within 72 hours of admission, which may introduce selection bias toward children treated in centers with timely MRI access or children already suspected of having complicated disease. The development setting therefore differs from hospitals with delayed MRI access, limited sedation capacity, or different referral patterns.

Third, microbiological, nutritional, local-examination, and treatment-related variables were incompletely captured. Recent antecedent infections or prior minor infections were not systematically recorded and may have influenced baseline inflammatory markers and the clinical presentation at admission. Pre-admission antibiotic exposure, culture timing, local treatment thresholds, radiology protocol differences, and center effects may have influenced both predictors and outcomes. Local examination findings, such as focal swelling, tenderness severity, range-of-motion limitation, or fluctuance, were not recorded in a standardized manner and therefore were not included in the model. Albumin may also reflect nutritional status, systemic inflammation, or both, and this retrospective dataset could not disentangle these mechanisms.

Fourth, abscess subtype distribution and abscess burden were not systematically quantified. In particular, size, volume, number, and detailed anatomic extent were not recorded in a standardized form. Because formal slice-level central radiologic reclassification was not performed, retrospective subtype frequencies could introduce misclassification and were therefore not reported. The binary MRI-defined abscess outcome is clinically practical but may obscure differences in abscess burden that could influence inflammatory response, surgical decision-making, and prognosis.

Fifth, repeat imaging timing and formal time-to-normalization of inflammatory markers were not uniformly abstracted. The descriptive management findings should therefore be interpreted as clinical context for the MRI-defined phenotype rather than as causal evidence linking the model predictors to treatment decisions.

Sixth, internal validation, calibration plots, and decision curve analysis cannot establish transportability. Prospective external validation with standardized MRI phenotyping, prespecified abscess-burden measurements, and broader microbiological ascertainment is required before clinical application.

## 5 Conclusions

In conclusion, lower admission albumin and higher admission CLR were associated with MRI-defined abscess in pediatric AHO. Together with fever at admission, these routinely available variables formed a parsimonious model with modest internally validated discrimination, supporting their potential value for early recognition of children who may benefit from prioritized MRI or multidisciplinary review. The model should complement clinical assessment and imaging decisions rather than function as a stand-alone diagnostic rule. Prospective external validation in larger multicenter cohorts is required before implementation.

## Data Availability

The raw data supporting the conclusions of this article will be made available by the authors, without undue reservation.
